# Financial toxicity and strain among men receiving prostate cancer care in an equal access healthcare system

**DOI:** 10.1002/cam4.3484

**Published:** 2020-10-17

**Authors:** Alexandria G. Bauer, Melanie Jefferson, Georges J. Nahhas, Stephen Savage, Richard Drake, Michael Lilly, Linda Ambrose, Susan Caulder, David Mahvi, Chanita Hughes Halbert

**Affiliations:** ^1^ Department of Psychiatry and Behavioral Sciences Medical University of South Carolina Charleston SC USA; ^2^ Hollings Cancer Center Medical University of South Carolina Charleston SC USA; ^3^ Department of Urology Medical University of South Carolina Charleston SC USA; ^4^ Ralph H. Johnson Veteran Affairs Medical Center Charleston SC USA; ^5^ Department of Cell and Molecular Pharmacology and Experimental Therapeutics Medical University of South Carolina Charleston SC USA; ^6^ Department of Medicine Medical University of South Carolina Charleston SC USA

**Keywords:** disparities, financial toxicity, healthcare system, prostate cancer

## Abstract

**Purpose:**

To examine financial toxicity and strain among men in an equal access healthcare system based on social determinants and clinical characteristics.

**Methods:**

Observational study among men receiving prostate cancer care (*n* = 49) at a Veterans Health Administration (VHA) facility. Financial hardship included overall financial strain and financial toxicity due to healthcare costs. Financial strain was measured with one item asking how much money they have leftover at the end of the month. Financial toxicity was measured with the Comprehensive Score for Financial Toxicity (COST) scale.

**Results:**

Comprehensive Score for Financial Toxicity scores among participants indicated moderate levels of financial toxicity (*M* = 24.4, SD = 9.9). For financial strain, 36% of participants reported that they did not have enough money left over at the end of the month. There were no racial or clinically related differences in financial toxicity, but race and income level had significant associations with financial strain.

**Conclusion:**

Financial toxicity and strain should be measured among patients in an equal access healthcare system. Findings suggest that social determinants may be important to assess, to identify patients who may be most likely to experience financial hardship in the context of obtaining cancer care and implement efforts to mitigate the burden for those patients.

## Graphical abstract

The purpose of this study was to examine financial toxicity and strain among men in an equal access healthcare system based on social determinants and clinical characteristics. There were no racial or clinically related differences in financial toxicity, but race and income level had significant associations with financial strain. Social determinants should be assessed to identify patients who may be most likely to experience financial hardship in the context of obtaining cancer care in order to implement efforts to mitigate the burden for those patients.

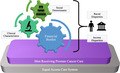

## 1 INTRODUCTION

Cancer is one of the leading causes of morbidity and mortality among adults in the United States^1^ and has significant financial consequences. In 2015, the direct costs for cancer care were as high as $80.2 billion, and out‐of‐pocket (OOP) costs for cancer patients were estimated to be as high as $4 billion.^1^, ^2^ Financial toxicity, or the extent to which patients experience financial problems as a result of costs for cancer treatment, is a local and national crisis, with implications for quality of life^2^ and health outcomes.^3^, ^4^, ^5^ Risk factors for financial toxicity include the type of cancer diagnosis and treatment.^3^ Social determinants (e.g., age, race/ethnic minority, income, health insurance coverage) are also associated with financial toxicity,^3^ meaning that some patients, particularly racial/ethnic minorities, may have experienced the burden of long‐term socioeconomic stressors (e.g., low income, unemployment) prior to beginning cancer care and incurring additional financial costs and economic hardship associated with receiving treatment.^6^, ^7^

Although financial toxicity has been documented in countries with universal healthcare programs,^8^, ^9^, ^10^, ^11^ it may be particularly severe for countries with individualized healthcare, such as the United States.^12^ The Veterans Affairs Health Care System (Veterans Health Administration, VHA) is one of the largest healthcare systems and insurers in the United States, and Veterans who use the VHA for their medical care are shown to have fewer socioeconomic resources and other social determinants that place them at greater risk for financial toxicity, compared to Veterans who do not use the VHA.^13^ Greater investigation into financial toxicity and identification of potential intervention targets^14^, ^15^ is now a priority at national and local levels. However, empirical data are not available on financial toxicity among patients in an equal access healthcare system such as the VHA.

The purpose of this exploratory study was to characterize financial toxicity and financial strain among patients receiving prostate cancer care at a VHA facility. We predicted that African American and minority Veterans would have greater financial toxicity and financial strain compared to white Veterans and that social determinants (e.g., low income) would also be significantly associated with these financial hardship variables. We also explored the relationship of clinical factors (e.g., co‐morbidity status) on financial hardship among these patients.

## 2 MATERIALS AND METHODS

### 2.1 Study sample

Participants were minority and non‐Hispanic white men who were scheduled to undergo a prostate biopsy because of an elevated prostate‐specific antigen (PSA) test, an abnormal digital rectal examination, or as part of active surveillance for low‐risk prostate cancer at the Urology Clinic at the Ralph H. Johnson Veteran's Affairs Medical Center (RHJ VAMC). Eligibility criteria included participants being male, between the ages of 40‐79 years old, and willing to allow researchers to follow their prostate cancer care (e.g., diagnosis, treatment decision, follow‐up care). Men who were unable or unwilling to provide informed consent were excluded from the study. A total of 124 men were identified for the study and invited to participate, of which 49 completed the survey. Minority men included participants who self‐reported their race as African American or another other minority group (e.g., Native American), or self‐reported Hispanic ethnicity.

### 2.2 Procedures

Patients were invited to participate in the study before undergoing their planned prostate biopsy between April 2017 and October 2018. Men undergoing prostate biopsy were included in this study because this diagnostic procedure is a critical clinical event in the trajectory of cancer diagnosis, treatment, and recovery. Following provision of written informed consent, information on race/ethnicity, age, co‐morbidities (e.g., hypertension, diabetes), and prostate cancer variables (e.g., PSA, number of positive biopsy cores) were abstracted from the electronic health record (EHR). Patients were also invited to complete a social determinants survey (SDS) following enrollment. Patients could decline to complete the SDS at the clinic visit, while interested patients were given the SDS to complete during their clinic visit or at home. For the purpose of this study, only participants who completed the SDS were included in analyses. This study was approved by the Institutional Review Board at the Medical University of South Carolina and the Veterans Affairs Research and Development Committee at the RHJ VAMC.

### 2.3 Measures

Social determinants (e.g., race/ethnicity, age, education, employment status, income level, and marital status) were measured using items from our previous research.^16^ We re‐coded these variables into conceptually meaningful dichotomous variables based on the distribution of responses. Clinical factors included co‐morbidity status and prostate cancer‐related outcomes. Co‐morbidity status was determined based on whether patients had a history of hypertension, diabetes, heart problems, stroke, or high cholesterol documented in their EHR. We created a dichotomous variable for co‐morbidity status that reflected whether patients had at least one co‐morbidity or did not have any co‐morbidities. Prostate cancer variables included PSA, the number of positive cores identified during the biopsy, and a personal history of prostate cancer.

Financial toxicity was measured using the Comprehensive Score for Financial Toxicity (COST) scale.^2^ The COST scale is an 11‐item Likert style instrument that was developed to measure the extent to which patients worry about their financial problems, are satisfied with their current financial situation, or feel financially stressed. Minor wording changes were made to some of the survey items to make them appropriate for the clinical context and where, men were in terms of their cancer trajectory. For instance, men were asked to indicate how much they worry about financial problems they will have as a result of their screening, illness, or treatment. After reverse scoring items that are positively worded, items were summed so that lower scores reflect greater financial toxicity. The COST scale had good internal consistency in our sample (Cronbach's *α* = 0.87). To measure financial strain, men were asked to indicate how much money they have left over at the end of the month (e.g., some money, just enough money, not enough money). This item was developed to measure perceptions of economic strain^17^, ^18^ and has acceptable face validity.^19^

### 2.4 Data analysis

First, descriptive statistics were generated to characterize participants in terms of social determinants, clinical factors, financial strain, and financial toxicity. Means and SDs were reported for continuous variables and frequencies and percentages were reported for dichotomous variables. Confidence intervals (95%) were generated for financial hardship variables. Next, bivariate analyses consisting of *t*‐test, chi‐square tests of association, and correlation analysis were conducted to examine relationships between financial hardship variables with social determinants and clinical factors.

## 3 RESULTS

Table 1 shows the characteristics of the study sample. Most participants were minority men, were married, and had at least some college education. The majority of participants had a personal history of prostate cancer, and 80% had at least one chronic condition (e.g., diabetes). Table 2 provides descriptive information on financial hardship variables. A one‐sample t‐test showed that participants experienced a significant level (*t* = 17.2, *p* < 0.001) of financial toxicity (*M* = 24.3, SD = 9.9 [95% CI = 21.4, 27.1]) compared to 0. A significant proportion of men (*z* = −1.90, *p* = 0.03 [95% CI = 22.7, 51.4]) also reported financial strain; 36% of men reported that they did not have enough money left over at the end of the month.

**TABLE 1:** Sample characteristics (*n* = 49)^a^

Variable	Level	*n* (%)
Race	Minority	27 (55)
	White	22 (45)
Marital status	Married	24 (55)
	Not married	20 (46)
Education level	≥Some college	27 (56)
	≤High school	21 (44)
Employment status	Employed	15 (33)
	Not employed	18 (39)
	Retired	13 (28)
Income level	>$35,000	17 (46)
	<$35,000	20 (54)
Medicare coverage	Yes	9 (18)
	No	40 (82)
Personal history prostate cancer	Yes	32 (65)
	No	17 (35)
Chronic disease	Yes	39 (80)
	No	10 (20)
Age	Mean (SD)	64.3 (7.3)

^a^ *n* may not equal sample size because of missing data.

**TABLE 2:** Descriptive information on financial toxicity and strain

Financial toxicity items	Mean	SD
I feel financially stressed	2.43	1.4
I am satisfied with my current financial situation	1.41	1.2
I worry about the financial problems I will have in the future as a result of my illness or treatment	2.35	1.4
I am frustrated that I cannot work or contribute as much as I usually do	2.35	1.4
My healthcare has reduced my satisfaction with my present financial situation	2.67	1.3
I feel in control of my financial situation	1.84	1.2
I am able to meet my monthly expenses	2.50	1.1
I know that I have enough money in savings, retirement, or assets to cover the costs of my healthcare	1.00	1.3
I am concerned about keeping my job and income, including working at home	2.76	1.4
I feel I have no choice about the amount of money I spend on care	2.41	1.4
My out‐of‐pocket medical expenses are more than I thought they would be	2.56	1.5
Mean	24.3	9.8

Financial strain	*n*	% Responding
Some money left over	22	47
Just enough money left over	8	17
Not enough money left over	17	36

Table 3 shows the results of the bivariate analysis of financial toxicity and financial strain. There were no racial differences in financial toxicity among participants, and none of the clinical factors were associated significantly with financial toxicity (e.g., *r* (PSA) = 0.005, *p* = 0.97 and *r* (number of positive cores) = 0.10, *p* = 0.50). Employment status approached significance (*p* = 0.06), with mean levels of financial toxicity being higher among men who were not employed and those who were employed compared to men who were retired. No other variables were associated with financial toxicity.

**TABLE 3:** Bivariate analysis of financial strain and toxicity

Variable	Level	Financial toxicity	*t*‐value	% Financial strain	Chi square
		Mean (SD)			
Race	Minority	23.8 (11.1)	0.39	48	3.94**
	White	24.9 (8.4)		20	
Marital status	Married	23.8 (11.9)	0.35	36	0.01
	Not married	24.9 (7.9)		35	
Education level	≥Some college	25.6 (10.9)	−1.10	31	0.98
	≤High school	22.4 (8.4)		45	
Employment status	Employed	23.8 (9.8)	3.09^a^	27	2.03
	Not employed	20.6 (9.2)		50	
	Retired	29.4 (10.4)		33	
Income level	>$35,000	27.6 (12.1)	−1.52	12	6.21*
	<$35,000	22.4 (8.4)		53	
Chronic disease	Yes	23.4 (10.1)	1.19	34	0.33
	No	27.6 (8.7)		44	
Personal Hx PrCa	Yes	23.6 (9.8)	0.57	31	1.05
	No	25.4 (10.1)		47	

^a^ *F*‐value is reported.

** *p* < 0.05;

* *p* < 0.01.

Race and income level were significantly associated with financial strain. Minority men were more likely to report financial strain compared to white men. Nearly half of minority men reported that they did not have enough money left over at the end of the month compared to one‐fifth of white men. In addition, men who had incomes less than $35,000 were more likely to report financial strain compared to those with higher incomes. No other variables were associated significantly with financial strain.

## 4 DISCUSSION

This is among the first studies to examine financial toxicity and strain among men receiving cancer care services in a US‐based equal access healthcare system. The VHA is one of the largest healthcare systems in the United States, which provides medical care to more than nine million Veterans annually.^8^ In this sample of Veterans who were receiving prostate cancer care services, there were moderate levels of financial toxicity and a substantial minority of men (36%) reported financial strain (e.g., did not have enough money left over at the end of the month). Mean levels of financial toxicity from healthcare expenses were comparable to those reported among cancer patients in previous research.^2^, ^20^, ^21^, ^22^, ^23^ These findings suggest that having access to healthcare services through an equal access system may not protect men from experiencing financial toxicity or strain. Furthermore, while the cost of cancer care can contribute to financial toxicity and strain, these concepts are more complex than simply coverage for care.^5^

Although we examined the relationship between social factors and clinical variables that have been associated with financial toxicity in previous reports,^3^, ^22^, ^23^, ^24^ we were unable to identify a robust set of variables that had significant independent associations with financial hardship in this study. For instance, while mean levels of financial toxicity were greater among unemployed and employed men compared to men who were retired, this association was not statistically significant. There were also no racial or income differences in financial toxicity, and financial toxicity did not differ by reported personal history of prostate cancer diagnosis. The lack of association between social risk factors and financial toxicity may be due to our modest sample size; however, the mean level for financial toxicity in our sample was similar to the levels reported among cancer patients in other reports.^2^, ^20^, ^21^, ^22^ Furthermore, race and income levels were associated significantly with financial strain in the bivariate analyses, and these findings are consistent with the results of previous research on financial toxicity.^3^, ^22^, ^23^, ^24^

Associations between social determinants with financial strain and toxicity may differ from findings reported in other studies because of conceptual distinctions between these variables.^10^, ^25^ Financial toxicity reflects the extent to which patients experience economic problems as a result of healthcare costs,^2^ whereas financial strain is experienced when one's overall expenses exceed his or her income and has adverse psychological effects.^17^, ^19^ As such, financial strain is a global measure of economic hardship, whereas financial toxicity reflects economic challenges that result specifically from healthcare costs. Patients in the VHA may be less vulnerable to financial toxicity based on social determinants and clinical factors because the VHA does not require eligible Veterans to pay premiums for ambulatory or inpatient medical care, prescription medication, and medical equipment.^26^, ^27^ At the same time, VHA patients may still be required to cover the cost of copayments for medications and those whose financial resources are above certain thresholds may have other copayments related to their medical care.^27^ Although there were no racial differences in income levels in our sample (data not shown), findings from a previous analysis of socioeconomic characteristics among older male Veterans demonstrated that 34% of African American Veterans between ages 62‐69 had a median family income that relied on Social Security, and African American Veterans had the lowest median household income compared to white and Hispanic Veterans, regardless of age group.^28^ African American Veterans also had lower employment, greater work‐limiting disability, and more poverty than white Veterans.^28^ This may explain why African American and minority men in this study were more likely than white men to report financial strain; however, future research is needed to examine the association between financial hardship, poverty, and disability status.

### 4.1 Limitations

In considering the results of this study, some limitations should be noted. First, our study data were collected in 2018 and is based on men who were at different points in the trajectory of prostate cancer care, and the observational nature of our study design does not allow us to determine causality with respect to social determinants and financial toxicity and strain. Therefore, prospective, longitudinal research is needed to determine the causal relationship between social determinants and financial strain and toxicity, especially as men move through different phases of prostate cancer diagnosis and treatment. It is also important for prospective studies to examine financial toxicity and strain among patients who have other types of cancer with different treatment costs, and to compare gender differences in the financial impact of cancer care.

Our modest sample size underscores the need to evaluate the distribution of financial strain and toxicity in larger samples of Veterans. The association between employment status and financial toxicity is particularly important to examine in larger samples of Veterans, because these variables may have an especially complex relationship among these patients. Beyond having pensions from their military service, some Veterans may also have other employee‐sponsored retirement benefits, in addition to Medicare. The 2014 Kaiser Family Foundation Study of retiree health benefits^29^ found that about one‐third of Medicare beneficiaries also had retiree health benefits and compared to other Medicare beneficiaries, those who also had retiree health coverage were most likely to be white, have higher incomes, and report that they are in better health.^29^ More recently, Narang and Nicholas found that Medicare beneficiaries who were newly diagnosed with cancer, but did not have any supplemental health insurance coverage, had the highest mean OOP expenses ($8115) relative to those who had employee‐sponsored insurance ($5,492) or had coverage through the VHA ($2367).^30^ At the same time, Gilligan et al^21^ found that among new cancer patients participating in the Health and Retirement Study from 1998‐2014, being retired was associated with an increased likelihood of depleting one's financial assets within 2 years of being diagnosed. Future research is needed to disentangle the effects of employment status on financial strain and toxicity among Veterans, in order to increase precision of understanding how income is related to financial hardship among men receiving prostate cancer care in equal access care systems.

Despite these potential limitations, our study extends previous research on financial toxicity among cancer patients in several important ways. Our findings demonstrate that an equal access care system may not protect men from experiencing financial toxicity and strain. The mean levels of financial toxicity were significantly different from zero and a substantial minority of men reported financial strain. Thus, it may still be necessary to provide financial assistance and education to VHA patients even though the majority of their medical care expenses may be covered through this healthcare system. Our findings suggest that it may be important to target these educational efforts and other strategies to racial minorities and Veterans who have incomes less than $35,000. To do this, however, it is first necessary to obtain data on social determinants and measure financial strain and toxicity using self‐reported data from patients. Financial strain, income, and education are among the social factors that should be recorded in the EHR,^31^ and our findings suggest that it may also be important to obtain employment status and income from patients to identify those who are at greatest risk for financial toxicity and strain.

### 4.2 Conclusion

It is crucial to understand the correlates of financial hardship, in order to increase our ability to address this burden among patients receiving cancer care. Recent research has shown that financial insolvency (e.g., bankruptcy) is associated with nonadherence to treatment recommendations^22^ and puts cancer patients at an increased risk for early mortality.^32^ Currently, efforts to address financial hardships among patients include establishing policies to limit the amount of OOP expenses paid by cancer patients and making evidence‐based decisions about recommendations for cancer therapies, so that patients are most likely to be financially responsible for treatments that have the greatest likelihood for success ^32^. Other efforts emphasize educating patients to help them understand the costs of their medical care and linking patients with financial counselors to assist them with obtaining supplemental support and resources to cover OOP expenses.^33^, ^34^ By increasing our understanding of financial hardship, healthcare providers may be better able to increase the precision of educational efforts about the financial costs of cancer care and improve the referral of patients to social services that provide financial support.

## CONFLICT OF INTEREST

The authors declare no conflicts of interest.

## AUTHORS' CONTRIBUTIONS

Alexandria G. Bauer, PhD: Writing, data analysis, review and editing. Melanie Jefferson, PhD: Project implementation, writing. Georges J. Nahhas, PhD, MPH: Writing, data analysis. Stephen Savage: Conceptualization, project implementation, review and editing. Richard Drake, PhD: Conceptualization, project implementation, review and editing. Michael Lilly, MD: Conceptualization, project implementation, review and editing. Linda Ambrose, RN: Conceptualization, project implementation, review and editing. Susan Caulder, RN: Conceptualization, project implementation, review and editing. David Mahvi, MD: Conceptualization, project implementation, review and editing. Chanita Hughes Halbert, PhD: Conceptualization, methodology, writing, project administration and funding acquisition.

## Data Availability

Author elects to not share data.
